# Epitope-mapping of the glycoprotein from Crimean-Congo hemorrhagic fever virus using a microarray approach

**DOI:** 10.1371/journal.pntd.0006598

**Published:** 2018-07-09

**Authors:** Amanda Fritzen, Christian Risinger, Gulay Korukluoglu, Iva Christova, Arina Corli Hitzeroth, Natalie Viljoen, Felicity Jane Burt, Ali Mirazimi, Ola Blixt

**Affiliations:** 1 Department of Chemistry, University of Copenhagen, Frederiksberg, Denmark; 2 Refik Saydam National Public Health Agency, Ankara, Turkey; 3 National Center of Infectious and Parasitic Diseases, Sofia, Bulgaria; 4 Division of Virology, Faculty of Health Sciences, University of the Free State, Bloemfontein, Republic of South Africa; 5 Division of Virology, NHLS Universitas, Bloemfontein, Republic of South Africa; 6 Folkhälsomyndigheten, Solna, Stockholm, Sweden; 7 Department for Laboratory Medicine, Karolinska Institute and Karolinska Hospital University, Solna, Sweden; 8 National Veterinary Institute, Uppsala, Sweden; NIAID Integrated Research Facility, UNITED STATES

## Abstract

Crimean-Congo hemorrhagic fever virus (CCHFV) causes severe acute human disease with lethal outcome. The knowledge about the immune response for this human health threat is highly limited. In this study, we have screened the glycoprotein of CCHFV for novel linear B-cell epitopic regions using a microarray approach. The peptide library consisted of 168 synthesized 20mer peptides with 10 amino acid overlap covering the entire glycoprotein. Using both pooled and individual human sera from survivors of CCHF disease in Turkey five peptide epitopes situated in the mucin-like region and GP 38 (G15-515) and G_N_ G516-1037 region of the glycoprotein were identified as epitopes for a CCHF immune response. An epitope walk of the five peptides revealed a peptide sequence located in the G_N_ region with high specificity and sensitivity. This peptide sequence, and a sequence downstream, reacted also against sera from survivors of CCHF disease in South Africa. The cross reactivity of these peptides with samples from a geographically distinct region where genetically diverse strains of the virus circulate, enabled the identification of unique peptide epitopes from the CCHF glycoprotein that could have application in development of diagnostic tools. In this study clinical samples from geographically distinct regions were used to identify conserved linear epitopic regions of the glycoprotein of CCHF.

## Author summary

Crimean-Congo hemorrhagic fever (CCHF) is a widespread disease caused by a tick-borne virus belonging to the genus *Orthonairovirus* of the *Nairoviridae* family. The virus is responsible for outbreaks of severe viral hemorrhagic fever with a case fatality rate of approximately 30%. The CCHF virus is transmitted to people either by tick bites or through contact with infected animal blood or tissues. A mouse brain-derived vaccine against CCHF has been developed (included in this study) and used on a small scale in eastern Europe. There is no safe and effective vaccine widely available for human use. Currently, there are a limited number of serological assays commercially available for testing for CCHFV specific IgG and IgM. There are enzyme linked immunosorbent assays (ELISA) and imunnofluorescent assays (IFA) designed for screening human samples for diagnostic purposes however they are not cost effective for surveillance studies. The limiting factor for the replication of these protocols in other laboratories is the availability of antigens and (where relevant) specified monoclonal antibodies. To contribute to the improvement of the diagnostic methods for CCHFV, we aimed to identify and characterize new synthetic antigens that were more sensitive and specific and could be applied in epidemiologic surveys.

## Introduction

Crimean-Congo hemorrhagic fever virus (CCHFV) is a tick-borne viral zoonosis distributed in Africa, Asia, eastern Europe and the Balkans. Ticks belonging to the genus *Hyalomma* are considered the principal vectors and the broad geographic distribution of the virus correlates with that of the vector [1,2]. More recently the virus has been identified as a cause of human disease in Spain [3], Greece [4], and India [5]. There is growing concern that the virus could emerge in other southern European countries which are within the distribution range of the vector [6,7]. The virus causes a disease that ranges in severity for reasons that are not clear [1, 2]. Fatality rates vary depending on the severity of the disease and can be as high as 30% in some countries.

Current diagnosis of CCHFV is based on the detection of viral RNA using RT-PCR, isolation of the virus and/or IgM/IgG detection [8,9]. Hence the detection of an antibody response against CCHFV is important for diagnosis as well as seroprevalence studies. Currently, most reagents used for development of diagnostic tools are dependent on culturing the virus within the confines of a biosafety level 4 facility. The biosafety considerations limit the number of laboratories which are able to prepare reagents [10]. The current perceived risk of spread of the virus to non-endemic regions highlights the importance of increasing diagnostic capacity and serological surveillance using safe, standardized reagents [9].

The viral genome consists of three RNA segments designated small (S), medium (M) and large (L). The S segment encodes the nucleocapsid, while the L segment translates into the RNA polymerase. The M segment encodes for a glycoprotein precursor that is post-translationally cleaved and generates mature G_N_ and G_C_ and a mucin-like domain [11]. Serological assays have been developed based on the recombinant nucleocapsid protein [8] but the use of glycoproteins has not been extensively investigated possibly due to the inherent challenges associated with preparing recombinant glycoproteins. However, peptides mimicking epitopic regions could have a potential as diagnostic tools and if the epitopes induce protective immunity they could play a role in vaccine development [12]. Goedhals *et al*. identified two possible epitopic regions in the G_C_ of CCHFV [13] which could have potential in further development of serological assays and warrant further investigation.

In this study we used a microarray technique creating a high throughput and cost effective method to screen B-cell peptide epitopes [14–16] covering the complete glycoprotein precursor of CCHFV. This is the first study using microarray technology to screen clinical samples from survivors of CCHF infections in South Africa and Turkey. This data was also further examined using serum samples from vaccinated individuals that received the Bulgarian vaccine. Interestingly, we have identified several specific peptide sequences which may have application in development of serological assays and also in vaccine development.

## Materials and methods

### Solid Phase Peptide Synthesis (SPPS)

Overlapping peptides representing the complete glycoprotein precursor of a strain of CCHFV including the O-glycosylated mucin-like domain and GP38 at amino acid positions 15–515, G_N_ and NS_M_ (515–1037) and G_C_ (1037–1688) (strain Turkey-Kelkit06, uniprot #C7F6X8) were prepared by a modified automated Fmoc-SPPS (Solid-Phase Peptide Synthesis) methodology on a Syro II peptide synthesizer (MultiSynTech, Witten, Germany) as described previously [17,18]. All samples were screened against a peptide library representing a CCHFV isolate from Turkey to identify conserved epitopic regions between different lineages.

### Printing of microarrays

Peptides were selectively enriched by covalent immobilization onto amine reactive N-hydroxy succinimide activated hydrogel coated MPX16 glass slides (Schott Nexterion, SlideH) with a BioRobotics MicroGrid II spotter (Genomics Solution) using Stealth 3B Micro Spotting Pins (ArrayIt) with approximately 6 nL per spot as described previously [17,18]. Printed glass slides were humidified for 30–60 min before N-hydroxy succinimide deactivation in blocking buffer (50 mM ethanolamine in 50 mM sodium borate, pH 8.5) for 30 min, then rinsed quickly in water and spun dry (VWR, Galaxy MiniArray). The blocked glass slides were fitted into superstructures; 2/16/48 well (FAST FRAME, Schleicher & Schuell (Whatman)) to make separate identical peptide libraries. To each library either 500/100/10 μL PLI-P (0.5 M NaCl, 3 mM KCl, 1.5 mM, KH_2_PO4, 6.5 mM Na_2_HPO4, pH 7.4, 3% bovine serum albumin (BSA) was added dependent on the superstructure.

### On-slide glycosylation of peptides

Blocked slides were fitted with a 2-well superstructure (FAST FRAME, Schleicher & Schuell (Whatman)) to form 2 wells. The wells were filled with 500 μL of glycosylation mixture (10 μL of 100 mM UDP-GalNAc (Sigma-Aldrich), 10 μL of either 0.36 mg/mL mM GalNAc-transferase 2 [GalNAc-T2] (SBH Biosciences) or 0.46 mg/mL GalNAc-transferase 3 [GalNAc-T3] (SBH Biosciences) 10 mM and 480 μL HEPES buffer, placed in a humidification chamber, and incubated for two hours at room temperature (RT). Slides were then washed with 0.1 M AcOH (2 x 5 min, shaking) and PLI-P (5 min, shaking). Slides were again washed with phospate buffered saline (PBS) pH7.4, rinsed thoroughly with water, dried by centrifugation, and were then ready to be immediately used in a subsequent lectin binding experiment [15,17]. Slides were incubated with biotinylated VVA lectin (1:500 dilution in PLI-P buffer) for 1 hour at RT, followed by incubation with streptavidin-AlexaFluor 647 (1:1000 dilution in PLI-P) for 1 hour.

### Immunscreening using peptide assays

Pooled (to conserve resources) or individual CCHFV IgG antibody positive sera and CCHFV IgG negative sera or serum samples IgG positive for varicella zoster virus (VZV), Epstein-Barr virus (EBV), herpes simplex virus (HSV) and tick-borne encephalitis (TBE) were diluted (1:10, 1:20, 1:25, 1:50) in incubation buffer PLI-P (0.5 M NaCl, 3 mM KCl, 1.5 mM KH_2_PO_4_, 6.5 mM Na_2_HPO_4_, pH = 7.4, 3% BSA), added directly onto slide subarrays and incubated for minimum 1 h (up to 2 days) on a shaking plate with a slow rotation. Slides were washed three times using PBS. Positive reactors were detected using goat anti-human IgG-Cy3 (Fc specific, 10μg/mL, Sigma-Aldrich) diluted (1:1000) in PLI-P. After the final wash, slides were spun dry and scanned followed by image analysis. Slides were scanned using a ProScanArray microarray scanner (Perkin Elmer) equipped with laser for excitation at 543 nm and images were analyzed with Scan Array Express software. Spots were identified using automated spot finding with manual adjustments for irregularities in print. The final data was obtained from the mean spot Relative Fluorescence Units (RFU) from all replicate spots for each sample (3 or 5 spots). Spot intensities were determined by subtracting the median pixel intensity of the local background from the average pixel intensity within the spot. The quality control covered intra- and interchip quality analysis of replicates. For the selected peptides, serum samples with relative fluorescent values higher than two standard deviations over the mean of the control group were designated as positive.

### Patient sera

The samples were routinely heated at 56°C for 1 hour prior to handling. All antibody positive samples used in the study were from convalescent or vaccinated individuals and hence would be negative for CCHFV.

#### Turkey

A total of 30 CCHF IgG positive sera from Refik Saydam National Public Health Agency, Ankara, Turkey were included in the study. All samples were confirmed CCHF IgG antibody positive using a commercial ELISA (Vektor-Best, Novosibirsk, Russia). Ethics approval was obtained for samples collected from patients in Turkey (Ethics approval number 2009–0728).

#### Bulgaria

The study included eight samples obtained from medical personnel who were voluntarily vaccinated in order to produce hyperimmune gamma-globulin. Of the ten participants, seven were female and three male, and their age ranged from 30 to 55 years (median age 48). The first group of four participants (individuals 1–4) had received several vaccinations and blood samples were taken one month after the last immunization. The second group of four participants (individuals 5–8) that had just completed a full vaccination program (consisting of three doses, plus one last dose one year after the first dose) and blood samples were taken one month after the last dose (Ethics approval number 2/2011).

#### South Africa

A total of 41 CCHF IgG positive sera from 14 laboratory confirmed patients in South Africa were included in the study. Serum samples were collected at intervals ranging from several months after infection up to 12 years after illness. All samples were confirmed CCHF IgG antibody positive using a commercial immunofluorescent antibody assay (EuroImmune). In addition, 11 CCHF IgG negative sera from 11 healthy volunteers were included as controls samples. Ethics approval was obtained for samples collected from patients in South Africa (Ethics Committee number 152/06).

#### Control sera

37 clinically validated sera from individuals infected with other viral diseases such as TBE, (n = 9), EBV, (n = 10) [16] and HSV, (n = 9) [15,19] and VZV, (n = 9) [19] were included in the study.

#### Ethical statement

Ethics approval was obtained for samples collected from patients in Turkey, Bulagria, South Africa (Ethics approval numbers 2009–0728 (Turkey), 2/2001 (Bulgaria)ECUFS 152/06 (South Africa). All participants (Turkey, Bulgaria and South Africa) were adults. Written informed consent was obtained from participants. All the collected samples were derived from blood donors and anonymized at the hospital/institute where the samples were originally collected, i.e. without possibility to trace samples back to the donor.

### Statistical analysis

All the data represented in this study are mean values from 3–5 replicates. The final data was obtained from the mean spot Relative Fluorescence Units (RFU) from all replicate spots for each sample (3 or 5 spots). Spot intensities were determined by subtracting the median pixel intensity of the local background from the average pixel intensity within the spot. The quality control covered intra- and interchip quality analysis of replicates. For the selected peptides, serum samples with relative fluorescent values higher than two standard deviations over the mean of the control group were designated as positive. A One-way-ANOVA (Prism Graphpad 7 software) was conducted as needed.

## Results

### Reactivity of serum samples collected from survivors in Turkey

**Fig 1:**
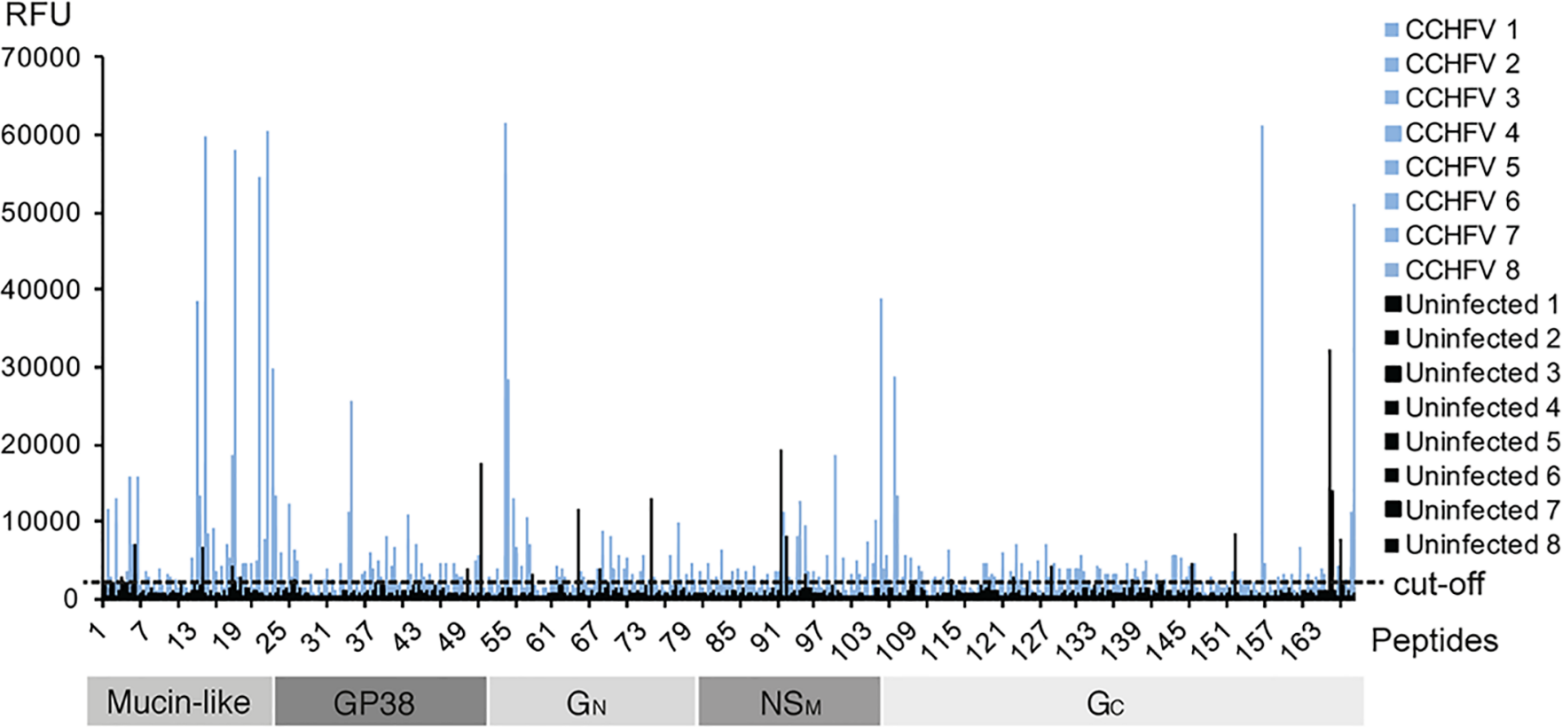
Reactivity of pooled Turkish sera. Reactivity of eight CCHFV survivor pools (blue) and six uninfected pools (black) against 168 peptides representing the complete precursor glycoprotein of CCHFV (Turkish strain, Kelkit06, Uniprot #C7F6X8).

A scan peptide library consisting of 168 peptides (20mer with a 10 amino acid overlap, see S1 Table) representing the glycoprotein precursor which includes the mucin-like domain, GP38, and G_N_ and G_C_ region of a strain of CCHFV from Turkey, was incubated with pooled inactivated sera from survivors of CCHFV infection. Reactivity against peptides representing possible epitopic regions were identified (Fig 1). Samples with a relative fluorescent value greater than two standard deviations above the mean of the control group were designated as positive.

Sera from survivors of CCHFV reacted significantly against fourteen peptides (entry 1–14, Table 1). Although there was minor reactivity from control sera against some peptides (entry 15–20, Table 1), the relative fluorescent values for these sera were significantly lower and these reactions were considered non-specific. From the results of this initial screen, 32 peptides were selected based on a combination of high reactivity of CCHFV positive sera and low reactivity of control sera (S2 Table) and narrowed down the selection to the five most promising peptide epitopes (entry 6, 7, 12, 13 and 14, (Table 1)). To further map the epitopes with respect to peptide sequence, a stepwise single-amino-acid epitope walk library of 20mers of these peptides was synthesized [15].

**Table 1:** Sequences of scan peptides that showed significant reactivity with pooled Turkish sera of CCHFV survivors (1–14) and with control sera (15–20). Five possible epitopes (6, 7, 12, 13 and 14) were selected for further evaluation using a single-amino acid epitope walk analysis.

ID	Peptide	Sequence
1	p14	^131^TSPSSSPSTPSTPQGIYHPA^150^
2	p15	^141^STPQGIYHPARSLLSVSSPK^160^
3	p19	^181^HSAMSRIPTPHTATRVSTEN^200^
4	p22	^211^SSAQQTTPSPMTSPAQSILL^230^
5	p23	^221^MTSPAQSILLMSAAPTAVQD^240^
6	p24	^231^MSA APTAVQDIHPSPTNRSK^250^
7	p55	^541^ETAEIHDDNYGGPGDKITIC^560^
8	p56	^551^GGPGDKITICNGSTIVDQRL^570^
9	p78	^771^RLTSDGLARHVTQCPKRKEK^790^
10	p96	^951^NVMLAVCKRMCFRATIEASR^970^
11	p99	^981^TTFVICILTLTICVVSTSAV^1000^
12	p105	^1041^RKPLFLDSIVKGMKNLLNST^1060^
13	p107	^1061^SLETSLSIEAPWGAINVQST^1080^
14	p156	^1551^PQSILIEHKGTIIGKQNDTC^1570^
15	p51	^501^SVLRQYKTEIKIGKASTGFR^520^
16	p64	^631^VLDACDSSCEVMIPKGTGDI^650^
17	p74	^731^QYRELKPQTCTICETAPVNA^750^
18	p91	^901^CSIGSVNGFEIESHKCYCSL^920^
19	p164	^1631^RRTRGLFKYRHLKDDEETGY^1650^
20	p165	^1641^HLKDDEETGYRRIIERLNSK^1660^

**Fig 2:**
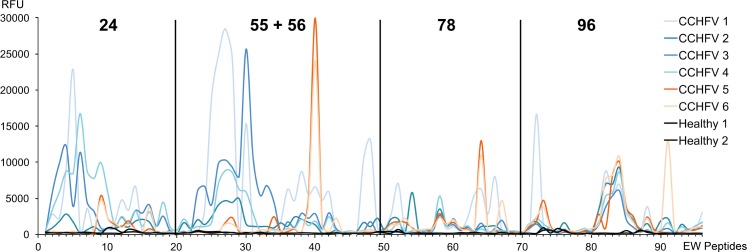
Epitope walk (EW) of selected five peptides (p24, p55, p56, p78 and p96). Figure illustrates the RFU of individual serum samples from CCHFV survivors from Turkey to selected peptides. Epitope walk sequences are found in S3 Table. In general two different patterns were seen when screening the epitopes (colored in blue and orange).

Six CCHFV IgG positive and two CCHFV IgG negative sera were analyzed individually, as shown in Fig 2. Overall, serum reactivities were high (>50%) against each peptide 20mer sequence. Two different reactivity patterns were apparent when screening the epitopes (colored in blue and orange in Fig 2). Four serum samples (designated CCHFV 1–4, blue lines) reacted strongly with early epitope walk (EW) peptides within libraries p24 and p55+56. In contrast, serum samples designated CCHFV 5 and 6 (orange) reacted weakly with these peptides, or not at all, and reacted strongly with peptide 40 within library p55+56 and peptide 64 in p78. The six serum samples reacted uniformly with p78 (EW 57–61) and p96 (EW 80–86). No reactivity was detected using the serum samples that were negative for CCHF IgG antibody (uninfected, black lines). High reactivity was seen against epitope walk (EW) residues 25–32 (p55+56) for four serum samples (CCHFV 1, 2, 3 and 4), whereas the remaining samples (orange) reacted against EW peptide 40 (p55+56). All sera reacted against EW peptides 81–86 within p96.

High reactivity was seen against epitope walk (EW) residues 25–32 (p55) for four serum samples CCHFV 1, 2, 3 and 4 (blue), whereas the remaining samples (orange) reacted against EW peptides 40 (p56). All sera reacted against EW peptides p81-86 derived from p96.

**Fig 3:**
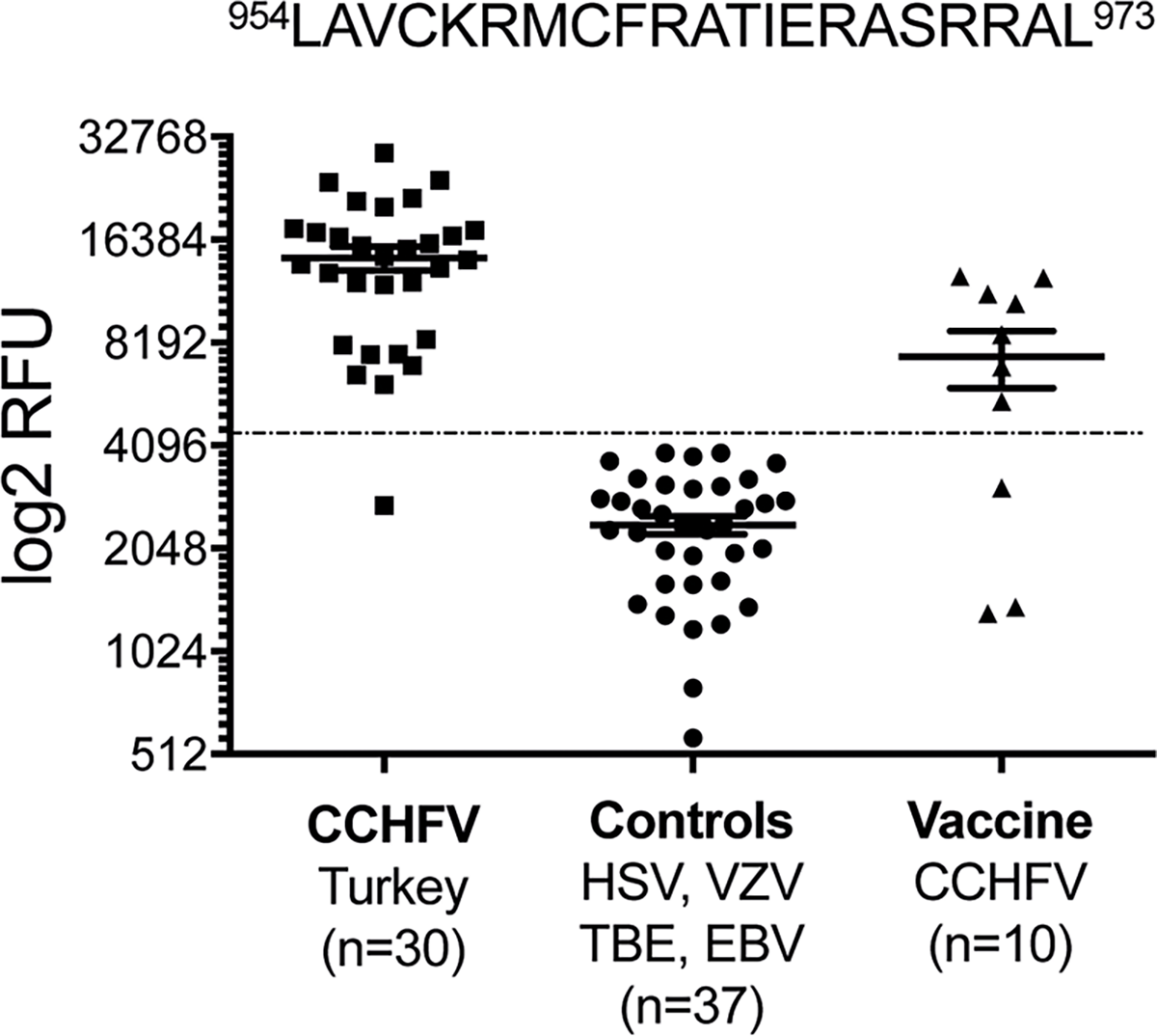
Dot-plot of intensities of the selected peptide EWP 83 (p96 + 3 aa, ^954^LAVCKRMCFRATIEASRRAL^973^) after serology. This includes sera from Turkish CCHFV survivors, control sera from individuals infected with other viral pathogens as controls and Bulgarian individuals vaccinated against CCHFV. The dotted line represents the diagnostic cut-off value determined from mean of the control group plus two standard deviations. P<0.0001.

Additional validation of p96 and its derived epitope walk peptides was performed with 30 sera from survivors of CCHFV from Turkey and 37 sera from individuals infected with other viral diseases such as TBE, VZV, EBV and HSV (Fig 3). The results demonstrate that the CCHFV infected individuals show high specificity and sensitivity (97%) to epitope p96. In addition, the activity of 10 sera from individuals vaccinated with the Bulgarian CCHFV vaccine [20] with p96 was analysed. This experiment also demonstrated that the vaccinated indivduals have significant reactivity towards this novel epitope p96 whereas the control sera (n = 37) showed no reactivity. The amini acid similarity between strains from Turkey and Bulgarian is high and the epitopic region identified has only minor differences which indicates that a similar reactivity towards this epitope could be expected. An analysis of variance showed that the reactivity of samples from Turkish survivors and from the vaccinated group relative to the control group was highly significant (F(2,74) = 68, P<0.0001).

Lastly, since glycosylation can be important for viral envelope proteins and inducing immune responses, microarray experiments were conducted to determine whether additional epitopes could be identified using glycosylated peptides. Peptides immobilised on slides were treated with ppGalNAc transferase 2 (T2) and ppGalNAc transferase 3 (T3), as described previously [15]. Glycosylation of peptides that represent parts of the mucin-domain were glycosylated as predicted with Net-O-Glyc (v3.1) algorithm. (S1 Fig). We did not observe any additional reactivity of the CCHFV sera after on-chip glycosylation of the peptides. However, one should have in mind that these data are based on peptide glycosylation experiments which may not mimic the natural glycosylation pattern on the envelop proteins.

### Reactivity of serum samples collected from survivors in South Africa

**Fig 4:**
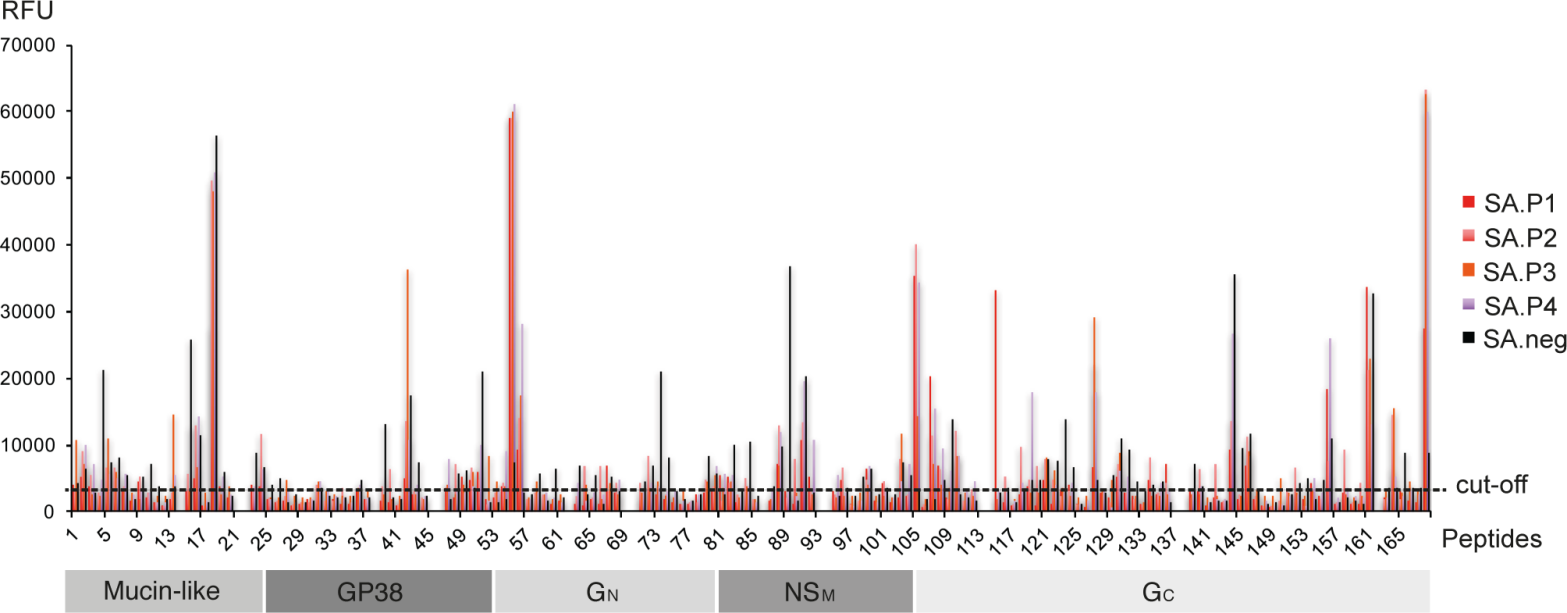
Reactivity of pooled South African sera. Four CCHFV survivor pools (red) and one uninfected pool (black)) against 168 peptides representing the complete precursor glycoprotein of CCHFV (Turkish strain).

To further evaluate the identified peptide epitopes, a cohort of sera from survivors of CCHFV in South Africa were screened for reactivity against the 168 20mer peptide library. A total of 41 CCHF IgG positive sera from 14 laboratory confirmed patients in South Africa were included in the study. All samples were confirmed to be CCHF IgG antibody positive using a commercial immunofluorescent antibody assay (EuroImmune). In addition, 11 CCHF IgG negative sera from 11 healthy volunteers were included as control samples. Pooled serum samples from confirmed patients reacted against peptides p55, p56, p105, p115, p119, p127 and p168 (Fig 4). Reactivity against peptides 119, 127 and 168 was unique to these sera.

**Fig 5:**
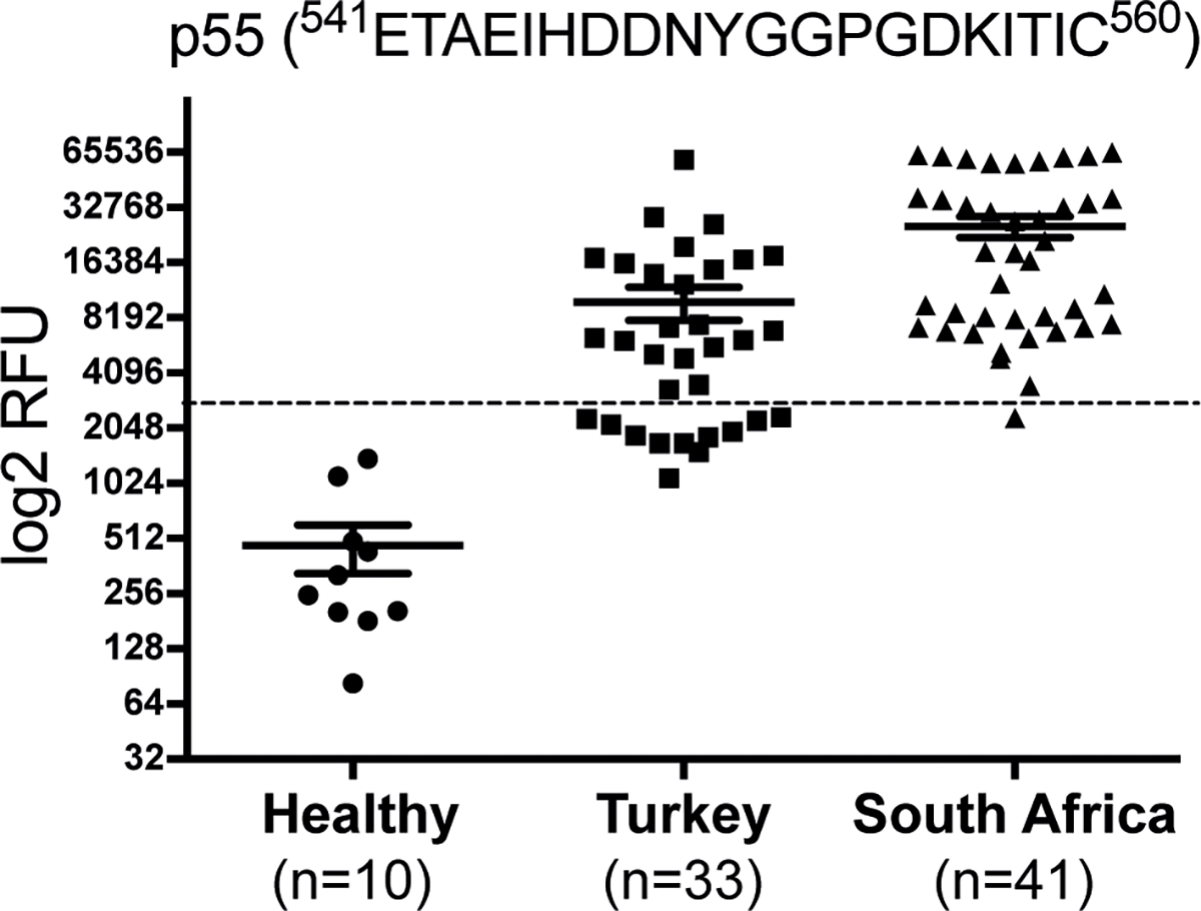
Dot-plot of intensities of the selected peptide 55 (^541^ETAEIHDDNYGGPGDKITIC^560^) after serology. This included sera from Turkish CCHFV survivors, control sera from individuals infected with other viral pathogens as controls and Bulgarian indivduals vaccinated against CCHFV. The dotted line represents the diagnostic cut-off value. P<0.0001.

Single serum sample experiments were performed using a 108 peptide sublibrary (see S1 Table, 1–108) from the larger 168 peptide library. As seen with the Turkish cohort of sera, the majority of the South African serum samples (97,6%) reacted against the p55 (ETAEIHDDNYGGPGDKITIC) (Fig 5). The specificity was even more pronounced for the South African sera. An analysis of variance showed that the reactivity of samples from Turkish survivors and SA survivors relative to the control group was highly significant (F(2,81) = 11, P<0.0001).

Nine peptides were identified which included a region that reacted against either Turkish or South African samples or reacted against both samples (Table 2). Finally the predicted amino acid sequences for each peptide region were retrieved from UniProt and aligned to identify similarities between the geographically distinct isolates of the virus (Table 3). This high coverage of same peptide reactivity can be explained by high amino acid conservation along the selected epitopes (Table 3). In contrast the alignment confirms up to four mismatches in the predicted amino acid sequence between the Turkish peptide sequence and the South African sequence for p96, thus possibly explaining their difference in reactivity of geographically diverse samples against this peptide.

**Table 2:** Reactivity of Turkish (n = 30) and South African sera (n = 41) against nine selected peptides.

Peptide	Negative^a^		Positive^b^		Positive x5^b^		Positive x10^b^		% Positive	
	Turkey	SA^c^	Turkey	SA	Turkey	SA	Turkey	SA	Turkey	SA
**24**	20	29	10	12	2	1	0	0	33	29
**24 EW27**	7		23		12		1		77	
**55**	12	1	18	40	10	22	3	18	60	**98**
**56**	16	17	14	24	6	5	3	2	47	59
**78**	27	15	3	26	1	3	0	0	10	63
**79**		25		16		10		6		39
**95**		26		15		0		0		37
**96**	23	33	7	8	0	0	0	0	23	20
**96 EW83**	1		29		0		0		**97**	
**96 EW84**	2		28		10		0		93	
**105**		14		27		15		5		66
**107**		30		11		3		0		27

^a^ values below 2 average of uninfected patient sera reactivity.

^b^ Positive x5/x10 indicated values 5/10 times above cut-off value.

^c^ South Africa

**Table 3:** Strain similarity, comparison of amino acid sequences from eight strains of CCHFV from South Africa and one strain of CCHFV from Turkey. Sequence data was retrieved from UniProt. Amino acid differences are bolded.

**Strain**	**Uniprot nr**	**p55**	**p56**	
**Turkey**	**C7F6X8**	**ETAEIHDDNYGGPGDKITIC**	**GGPGDKITICNGSTIVDQRL**	
**SA SPU431/85**	A0A068JCA2	ET**T**EIH**S**DNYGGPGDKITIC	GGPGDKITICNGSTIVDQRL	
**SA SPU383/87**	A0A068JFM1	E**N**AEIH**S**DNYGGPGDKITIC	GGPGDKITICNGSTIVDQRL	
**SA SPU130/89**	A0A068JC98	E**N**AEIH**S**DNYGGPGDKITIC	GGPGDKITICNGSTIVDQRL	
**SA SPU497/88**	A0A068JD49	E**N**AEIH**S**DNYGGPGDKITIC	GGPGDKITICNGSTIVDQRL	
**SA SPU45/88**	A0A068JCX3	E**NT**EIH**S**DNYGGPGDKITIC	GGPGDKITICNGSTIVDQRL	
**SA SPU187/90**	A0A068JCX6	ETAEIH**G**DNYGGPGDKITIC	GGPGDKITICNGSTIVDQRL	
**SA SPU48/90**	A0A068JD54	ETAEIH**G**DNYGGPGDKITIC	GGPGDKITICNGSTIVDQRL	
**Strain**	**Uniprot nr**	**p78**	**p96**	**p105**
**Turkey**	**C7F6X8**	**RLTSDGLARHYTQCPKRKEK**	**NVMLAVCKRMCFRATIEASRRALLIR**	**RKPLFLDSIVKGMKNLLNST**
**SA SPU431/85**	A0A068JCA2	RLTSDGLARH**VM**QCPKRKEK	NVMLAVCKRMCFRATIE**V**S**N**RALLIR	RKPLFLDSIVKG**R**KNLLNST
**SA SPU383/87**	A0A068JFM1	RLTSDGLARH**VI**QCPKRKEK	NVMLAVCKRMCFRAT**V**E**V**S**NK**ALLIR	RKPLFLDSIVKGMKNLLNST
**SA SPU130/89**	A0A068JC98	RLTSDGLARH**VM**QCPKRKEK	NVMLAVCKRMCFRAT**V**E**V**S**NK**ALLIR	RKPL**S**LDSIVKGMKNLLNST
**SA SPU497/88**	A0A068JD49	RLTSDGLARH**VM**QCPKRKEK	NVMLAVCKRMCFRAT**V**E**V**S**NK**ALLIR	RKPLFLDSIVKGM**R**NLLNST
**SA SPU45/88**	A0A068JCX3	RLTSDGLARH**VM**QCPKRKEK	NVMLAVCKRMCFRAT**M**E**V**S**SK**ALLIR	RKPLFLDSIVKG**K**KNLLNST
**SA SPU187/90**	A0A068JCX6	RLTSDGLARH**VT**QCPKRKEK	NVMLAVCKRMCFRAT**M**E**V**S**N**RAL**F**IR	R**R**PLFLDSIVKGMKNLLNST
**SA SPU48/90**	A0A068JD54	RLTSDGLARH**VT**QCPKRKEK	NVMLAVCKRMCFRAT**M**E**V**S**D**RAL**F**IR	RKPLF**R**DSIVKGMKNLLNST

**Fig 6:**
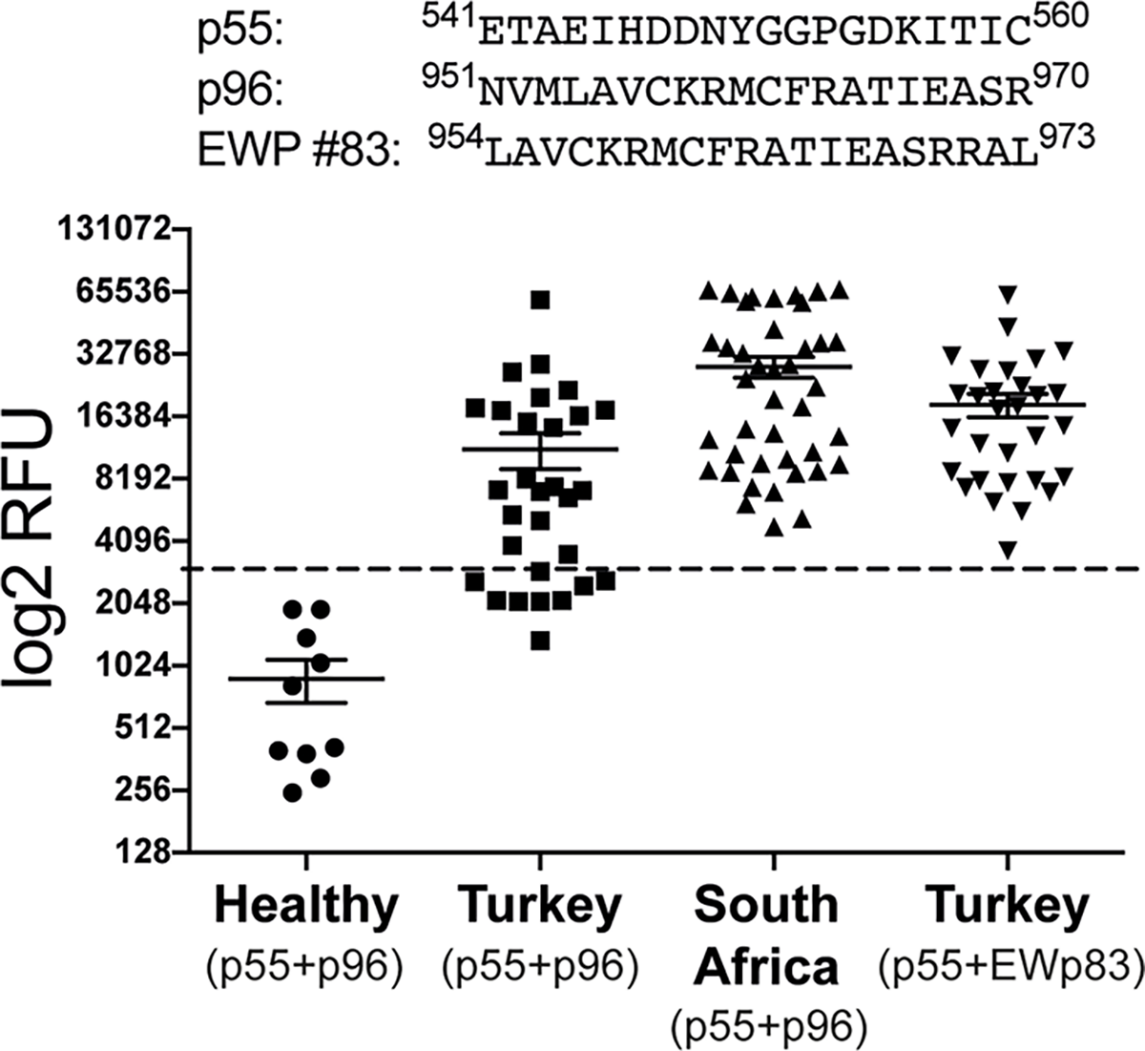
Dot-plot after combining intensities of serology with peptide p55 and p96 or EWP p83 (p96 + 3 aa). Control sera from healthy individuals, Turkish CCHFV survivors and South African CCHFV survivors. The dotted line represents the diagnostic cut-off value. P<0.0001.

The combination of p55/p56 (ETAEIHDDNYGGPGDKITIC/GGPGDKITICNGSTIVDQRL) with p96 (NVMLAVCKRMCFRATIEASR) represents an immunodominant epitopic region that would result in a multiepitope covering the majority of the Turkish and South African strains, due to minimal differences in the amino acid sequence along the defined epitopes, defined (Fig 6). An analysis of variance showed that the reactivity of samples from Turkish survivors and SA survivors and relative to the control group was highly significant (F(3,107) = 11, P<0.0001).

## Discussion

CCHFV is considered an emerging pathogen particularly in southern and eastern European countries. The emergence of this virus has significant public health implications. Currently there are a limited number of laboratories that can prepare reagents for diagnosis and serological surveillance as culturing the virus requires maximum containment facilities. Development of safe reagents will play a role in building capacity for diagnosis and surveillance. Development of reagents must take into consideration the global diversity of CCHFV. In this study peptides were used to identify potential epitopic regions on the glycoprotein precursor of an isolate from Turkey.

The initial screen was peformed using a total of 168 peptides representing the entire GP of a Turkish strain of CCHFV. Five peptides were selected for further investigation using epitope walk analysis in an attempt to identify specific peptide sequence mimicking immunodominant linear epitopes. Despite some variability in the reactivity of the samples from survivors in Turkey, common regions were identified within p96 and p55+56. Variability in the responses was particularly noted against the peptides of p24. The differentiated response to the EW peptides of p24 is most likely caused by the fact that p24 is located in the mucin-like region of the envelope protein which is highly glycosylated. The glycosylation of the mucin-like region can differ and it is therefore expected that the serum response to the naked peptides will be diverse [16]. The role of the mucin-like domain in stimulation of B cells during CCHF infections has not been well defined. By analogy with Ebola virus the mucin-like domain may block access to GP and actually inhibit immune responses [21]. However as the mucin-like domain is not incorporated into viral particles its role may be very different to that proposed for Ebola [11]. It would be useful to determine if the mucin-like domain is involved in immune evasion and if deletion of this region promotes an immune response to more conserved regions on the GP as has been shown for Ebola virus. This would be significant for vaccine development but less important for developing tools for detection of antibody responses.

In all the experiments p55 was identified as the top candidate due to the strong response shown when incubated with serum from CCHFV infected patients. The experiments with pooled sera showed a very dominating RFU signal, thereby undermining some of the other binding when reading the scan. The reaction towards p55 was scattered with around 2/3 of the samples binding to the peptide significantly where minor to no binding was seen in other serum samples. In the second group of sera (orange pattern) the highest reaction was shifted by 10 aa residues towards p56 in comparison to the blue pattern. A small common epitope was identified in p78 (EW 57–61) with all serum samples showing similar reactivity. This epitope could be of potential interest due to the homogenous binding. The response to this epitope walk was lower than as seen with the other epitope walks except for CCHFV 5 and 6 which reacted to EW peptide 64. The epitope with the strongest homogenous response was found in p96 epitope walk representing sequences being close to the transmembrane region (aa 973–997), and the tertiary structure can be expected to be more stable and should be in reach of B-cells/antibodies/immune cells. All serum samples tested showed binding towards the peptides EW peptides 80–86.

In summary, single amino acid shifts generated differentiation in reactivity patterns with no major reactive single peptide epitope for p24, p55, p56 and p78, whereas library members derived from p96 (epitope walk peptides p81-86, ^951^NVMLAVCKRMCFRATIEASRRALLIR^975^) show reactivity with all six CCHFV sera. No reactivity towards these peptides was observed with the two control sera.

Identification of peptides that are cross reactive serologically against geographically distinct strains is important for development of standardised assays for detection with application on different continents. Despite some variablity in the reactivity of samples from South African patients, a commonality was identified in p55.

The result of the epitope walk indicated that a strong differentiated binder could be obtained by combining the epitope ^541^ETAEIHDDNYGGPGDKITIC^560^ due to the strong signal of p55, with p96 (^951^NVMLAVCKRMCFRATIEASR^970^) for specific overall coverage. A combinational multi-epitope including these peptides could eventually secure a strong selectivity and sensitivity.

The study identified several specific peptide sequences which may have application in development of serological assays and possibly vaccine development. However for vaccine development it must be taken into consideration that the immune correlates of protection for CCHFV are currently not well defined and the role of T cells and discontinuous B cell epitopes would need to be considered. Evidence exists for a role for both antibody and T cell responses. A long lived cytotoxic T cell response was recently described in survivors of infection suggesting a role for T cells in protection [22]. However vaccine studies have suggested a role for both humoral responses and T cell responses. Results from a candidate vaccine employing a modified vaccinia virus Ankara poxvirus vector containing the GP in which passive and adoptive transfer of serum samples and T-lymphocytes were used in an attempt to define the role of each arm of the adaptive immune response, concluded that protective immunity likely requires both humoral and cellular involvement [23]. Serum samples collected from volunteers vaccinated using the inactivated Bulgarian vaccine reacted similarly to survivors of virus infection from Turkey with regard to peptides recognised although there were differences in intensity of reactivity. Sera from vaccinated individuals reacted with lower intensity than sera from naturally infected survivors. Previous investigations of immune responses in vaccinated individuals suggested that even after several boosters the neutralising antibody response was low. Although it is not known if the epitopic regions identified in this study represent neutralising epitopes it is possible that the lower reactivity is a reflection of a less robust immune response in vaccinees compared with survivors.

The use of glycoproteins has not been extensively investigated for serological assays likely due to the inherent challenges associated with expression of recombinant glycoprotein antigens. NP has been investigated as a target for diagnostic proteins due to its immunogenicty, abundance and homology between isolates. However although there is less sequence homology in the M gene compared to the S gene encoding for NP, a significant amount of the diversity is located within the mucin-like region with less than 10% amino acid diversity determined for the remainder of the GP [24]. It is probable that epitopes inducing immunodominant and/or neutralising responses are more likely to be conserved between isolates and in the absence of detailed data regarding immunodominant epitopes in the GP it is appropriate to further define if there are conserved regions with possible roles in development of diagnostic tools and incorporation in vaccine design. Peptides mimicking epitopic regions could have a potential as diagnostic tools [12]. Goedhals *et al*. identified two possible epitopic regions in the G_C_ of CCHFV and although in this study reactivity was identified in these epitopic regions using samples from South African survivors there was no significant reactivity from Turkish sera [13]. This highlights the need to consider diversity when selecting peptides to mimic epitopic regions.

Differences in serological reactivity between Turkish and South African samples suggested possible differences in protein sequences which were confirmed by alignment of predicted amino acid sequences. Hence the use of multiple peptides in downstream assay development will likely increase the usefulness of the assays. Lack of reactivity against serum samples collected from patients with other infectious diseases that could be considered in a differential diagnosis suggest that these peptides react more specifically against CCHFV antibodies. However the specificity and sensitivity will need to be validated in any future assay development. The cross-reactivity of these peptides with samples from a geographically distinct region where genetically diverse strains of the virus circulate enabled the identification of unique peptide epitopes from the CCHFV glycoprotein that could have application in develoment of diagnostic tools such as lateral flow or ELISA for antibody detection.

## Supporting information

S1 Table Related to Fig 1 shows CCHFV GN, GC and a mucin-like domain scan peptides prepared with Fmoc-SPPS used to produce microarray data. (DOCX)

S2 Table Sequences of 32 selected scan peptides in the first round that showed significant reactivity with pooled Turkish sera of CCHFV survivors. (DOCX)

S3 Table Related to Fig 2 –sequences of peptides synthesized for further epitope mapping of scan peptides p24, p55, p56, p78 and p96. (DOCX)

S1 Fig On-slide enzymatic O-glycosylation of scan peptides (20mer with 10mer overlap) with recombinant GalNAcT2 and GalNAcT3. (DOCX)
