# Supplementary material for: Epitope-mapping of the glycoprotein from Crimean-Congo hemorrhagic fever virus using a microarray approach
Source: PLoS Negl Trop Dis. 2018 Jul 9;12(7):e0006598. doi: 10.1371/journal.pntd.0006598 (PMC6053253; doi:10.1371/journal.pntd.0006598)
Supplement: S1 Table — (DOCX) [file pntd.0006598.s001.docx]

Supporting information

S1 Table, related to Figure 1: CCHFV G_N_, G_C_ and a mucin like domain scan peptides prepared with Fmoc-SPPS used to produce microarray data. All structures were prepared by means of automated parallel peptide synthesizer (for experimental procedures see methods section).

|  | 20mer  aa stretch | TURKEY C7F6X8  scan peptide sequence |  | 20mer  aa stretch | | TURKEY C7F6X8  scan peptide sequence |
| --- | --- | --- | --- | --- | --- | --- |
| 1 | 1-20 | MPTNIMHTLLVCFILYLQLL | 44 | | 431-450 | LSNCHVVINSHVCDYSLDTD |
| 2 | 11-30 | VCFILYLQLLCLGGAHGQLN | 45 | | 441-460 | HVCDYSLDTDGPVRLPRIYH |
| 3 | 21-40 | CLGGAHGQLNTTEHNGTNNT | 46 | | 451-470 | GPVRLPRIYHEGTFIPGTYK |
| 4 | 31-50 | TTEHNGTNNTTAPGASQSPK | 47 | | 461-480 | EGTFIPGTYKIVIDKKNKLN |
| 5 | 41-60 | TAPGASQSPKPPMSTTPPHA | 48 | | 471-490 | IVIDKKNKLNDRCTLVTNCV |
| 6 | 51-70 | PPMSTTPPHAPESSTIKPTT | 49 | | 481-500 | DRCTLVTNCVIKGREVRKGQ |
| 7 | 61-80 | PESSTIKPTTPISEAEGSGE | 50 | | 491-510 | IKGREVRKGQSVLRQYKTEI |
| 8 | 71-90 | PISEAEGSGETTSTPNTTQG | 51 | | 501-520 | SVLRQYKTEIKIGKASTGFR |
| 9 | 81-100 | TTSTPNTTQGLSSPETTSER | 52 | | 511-530 | KIGKASTGFRKLLSEEPGDD |
| 10 | 91-110 | LSSPETTSERPATTAISTSS | 53 | | 521-540 | KLLSEEPGDDCISRTQLLRT |
| 11 | 101-120 | PATTAISTSSTDSTNPTTQM | 54 | | 531-550 | CISRTQLLRTETAEIHDDNY |
| 12 | 111-130 | TDSTNPTTQMTDNTPTLTVS | 55 | | 541-560 | ETAEIHDDNYGGPGDKITIC |
| 13 | 121-140 | TDNTPTLTVSTSPSSSPSTP | 56 | | 551-570 | GGPGDKITICNGSTIVDQRL |
| 14 | 131-150 | TSPSSSPSTPSTPQGIYHPA | 57 | | 561-580 | NGSTIVDQRLGSELGCYTIN |
| 15 | 141-160 | STPQGIYHPARSLLSVSSPK | 58 | | 571-590 | GSELGCYTINRVKSFKLCEN |
| 16 | 151-170 | RSLLSVSSPKTVTTPTPTSP | 59 | | 581-600 | RVKSFKLCENSATGKTCEVD |
| 17 | 161-180 | TVTTPTPTSPGEMSSETSSQ | 60 | | 591-610 | SATGKTCEVDSTPVKCRQGF |
| 18 | 171-190 | GEMSSETSSQHSAMSRIPTP | 61 | | 601-620 | STPVKCRQGFCLKITQEGRG |
| 19 | 181-200 | HSAMSRIPTPHTATRVSTEN | 62 | | 611-630 | CLKITQEGRGHVKLSRGSEV |
| 20 | 191-210 | HTATRVSTENTNHSTPRQSE | 63 | | 621-640 | HVKLSRGSEVVLDACDSSCE |
| 21 | 201-220 | TNHSTPRQSESSAQQTTPSP | 64 | | 631-650 | VLDACDSSCEVMIPKGTGDI |
| 22 | 211-230 | SSAQQTTPSPMTSPAQSILL | 65 | | 641-660 | VMIPKGTGDILVDCSGGQQH |
| 23 | 221-240 | MTSPAQSILLMSAAPTAVQD | 66 | | 651-670 | LVDCSGGQQHFLKDNLIDLG |
| 24 | 231-250 | MSAAPTAVQDIHPSPTNRSK | 67 | | 661-680 | FLKDNLIDLGCPHIPLLGKM |
| 25 | 241-260 | IHPSPTNRSKRNLETEIILT | 68 | | 671-690 | CPHIPLLGKMAIYICRMSNH |
| 26 | 251-270 | RNLETEIILTLSQGLKKYYG | 69 | | 681-700 | AIYICRMSNHPRTTMAFLFW |
| 27 | 261-280 | LSQGLKKYYGKILKLLHLTL | 70 | | 691-710 | PRTTMAFLFWFSFGYVITCI |
| 28 | 271-290 | KILKLLHLTLEEDTEGLLEW | 71 | | 701-720 | FSFGYVITCISCKALFYSLI |
| 29 | 281-300 | EEDTEGLLEWCKRNLGSNCD | 72 | | 711-730 | SCKALFYSLIIIGTLGKKIK |
| 30 | 291-310 | CKRNLGSNCDDDFFQKRIEE | 73 | | 721-740 | IIGTLGKKIKQYRELKPQTC |
| 31 | 301-320 | DDFFQKRIEEFFMTGEGYFN | 74 | | 731-750 | QYRELKPQTCTICETAPVNA |
| 32 | 311-330 | FFMTGEGYFNEVLQFKTLST | 75 | | 741-760 | TICETAPVNAIDAEMHDLNC |
| 33 | 321-340 | EVLQFKTLSTLSPTEPSHAR | 76 | | 751-770 | IDAEMHDLNCSYNICPYCAS |
| 34 | 331-350 | LSPTEPSHARLPTAEPFKSY | 77 | | 761-780 | SYNICPYCASRLTSDGLARH |
| 35 | 341-360 | LPTAEPFKSYFAKGFLSIDS | 78 | | 771-790 | RLTSDGLARHVTQCPKRKEK |
| 36 | 351-370 | FAKGFLSIDSGYFSAKCYPR | 79 | | 781-800 | VTQCPKRKEKVEETELYLNL |
| 37 | 361-380 | GYFSAKCYPRSSASGLQLIN | 80 | | 791-810 | VEETELYLNLERIPWIVRKL |
| 38 | 371-390 | SSASGLQLINVTQHPARIAE | 81 | | 801-820 | ERIPWIVRKLLQVSESTGVA |
| 39 | 381-400 | VTQHPARIAETPGPKTTSLK | 82 | | 811-830 | LQVSESTGVALKRSSWLIVL |
| 40 | 391-410 | TPGPKTTSLKTINCINLRAS | 83 | | 821-840 | LKRSSWLIVLLVLLTVSLSP |
| 41 | 401-420 | TINCINLRASVFKEHREIEI | 84 | | 831-850 | LVLLTVSLSPVQSAPVGHGK |
| 42 | 411-430 | VFKEHREIEINVLLPQIAVN | 85 | | 841-860 | VQSAPVGHGKTIEIYQTREG |
| 43 | 421-440 | NVLLPQIAVNLSNCHVVINS | 86 | | 851-870 | TIEIYQTREGFASICLFMLG |
| 87 | 861-880 | FASICLFMLGSILFIVSCLV | 133 | | 1321-1340 | DLQVYHIGNLLKGDRVNGHL |
| 88 | 871-890 | SILFIVSCLVKGLVDSVSDS | 134 | | 1331-1350 | LKGDRVNGHLIHKIESHFNT |
| 89 | 881-900 | KGLVDSVSDSFFPGLSVCKT | 135 | | 1341-1360 | IHKIESHFNTSWMSWDGCDL |
| 90 | 891-910 | FFPGLSVCKTCSIGSVNGFE | 136 | | 1351-1370 | SWMSWDGCDLDYYCNMGDWP |
| 91 | 901-920 | CSIGSVNGFEIESHKCYCSL | 137 | | 1361-1380 | DYYCNMGDWPSCTYTGVTQH |
| 92 | 911-930 | IESHKCYCSLFCCPYCRHCS | 138 | | 1371-1390 | SCTYTGVTQHNHAAFVNLLN |
| 93 | 921-940 | FCCPYCRHCSADREIHQLHL | 139 | | 1381-1400 | NHAAFVNLLNIETDYTKTFH |
| 94 | 931-950 | ADREIHQLHLSICKKRKTGS | 140 | | 1391-1410 | IETDYTKTFHFHSKRVTAHG |
| 95 | 941-960 | SICKKRKTGSNVMLAVCKRM | 141 | | 1401-1420 | FHSKRVTAHGDTPQLDLKAR |
| 96 | 951-970 | NVMLAVCKRMCFRATIEASR | 142 | | 1411-1430 | DTPQLDLKARPTYGAGEITV |
| 97 | 961-980 | CFRATIEASRRALLIRSIIN | 143 | | 1421-1440 | PTYGAGEITVLVEVADMELH |
| 98 | 971-990 | RALLIRSIINTTFVICILTL | 144 | | 1431-1450 | LVEVADMELHTKKVEISGLK |
| 99 | 981-1000 | TTFVICILTLTICVVSTSAV | 145 | | 1441-1460 | TKKVEISGLKFASLACTGCY |
| 100 | 991-1010 | TICVVSTSAVEMENLPAGTW | 146 | | 1451-1470 | FASLACTGCYACSSGISCKV |
| 101 | 1001-1020 | EMENLPAGTWEREEDLTNFC | 147 | | 1461-1480 | ACSSGISCKVRIHVDEPDEL |
| 102 | 1011-1030 | EREEDLTNFCHQECQVTETE | 148 | | 1471-1490 | RIHVDEPDELTVHVKSSDPD |
| 103 | 1021-1040 | HQECQVTETECLCPYEALVL | 149 | | 1481-1500 | TVHVKSSDPDVVAASTSLMA |
| 104 | 1031-1050 | CLCPYEALVLRKPLFLDSIV | 150 | | 1491-1510 | VVAASTSLMARKLEFGTDST |
| 105 | 1041-1060 | RKPLFLDSIVKGMKNLLNST | 151 | | 1501-1520 | RKLEFGTDSTFKAFSAMPKT |
| 106 | 1051-1070 | KGMKNLLNSTSLETSLSIEA | 152 | | 1511-1530 | FKAFSAMPKTSLCFYIVERE |
| 107 | 1061-1080 | SLETSLSIEAPWGAINVQST | 153 | | 1521-1540 | SLCFYIVEREYCKSCSEDDT |
| 108 | 1071-1090 | PWGAINVQSTFKPAVSTANI | 154 | | 1531-1550 | YCKSCSEDDTQKCVDTRLEQ |
| 109 | 1081-1100 | FKPAVSTANIALSWSSVEHR | 155 | | 1541-1560 | QKCVDTRLEQPQSILIEHKG |
| 110 | 1091-1110 | ALSWSSVEHRGNKILVTGRS | 156 | | 1551-1570 | PQSILIEHKGTIIGKQNDTC |
| 111 | 1101-1120 | GNKILVTGRSESIMKLEERT | 157 | | 1561-1580 | TIIGKQNDTCTAKASCWLES |
| 112 | 1111-1130 | ESIMKLEERTGVSWDLGVED | 158 | | 1571-1590 | TAKASCWLESVKSFFYGLKN |
| 113 | 1121-1140 | GVSWDLGVEDASESKLLTVS | 159 | | 1581-1600 | VKSFFYGLKNMLGSVFGNLF |
| 114 | 1131-1150 | ASESKLLTVSIMDLSQMYSP | 160 | | 1591-1610 | MLGSVFGNLFIGILLFLAPF |
| 115 | 1141-1160 | IMDLSQMYSPVFEYLSGDRQ | 161 | | 1601-1620 | IGILLFLAPFVLLVLFFMFG |
| 116 | 1151-1170 | VFEYLSGDRQVEEWPKATCT | 162 | | 1611-1630 | VLLVLFFMFGWKILFCFKCC |
| 117 | 1161-1180 | VEEWPKATCTGDCPERCGCT | 163 | | 1621-1640 | WKILFCFKCCRRTRGLFKYR |
| 118 | 1171-1190 | GDCPERCGCTSSTCLHKEWP | 164 | | 1631-1650 | RRTRGLFKYRHLKDDEETGY |
| 119 | 1181-1200 | SSTCLHKEWPHSRNWRCNPT | 165 | | 1641-1660 | HLKDDEETGYRRIIERLNSK |
| 120 | 1191-1210 | HSRNWRCNPTWCWGVGTGCT | 166 | | 1651-1670 | RRIIERLNSKKGKNRLLDGD |
| 121 | 1201-1220 | WCWGVGTGCTCCGVDVKDLF | 167 | | 1661-1680 | KGKNRLLDGDRLADRKIAEL |
| 122 | 1211-1230 | CCGVDVKDLFTDHMFVKWKV | 168 | | 1669-1688 | GDRLADRKIAELFSTKTHIG |
| 123 | 1221-1240 | TDHMFVKWKVEYIKTEAIVC |  | |  |  |
| 124 | 1231-1250 | EYIKTEAIVCVELTSQERQC |  | |  |  |
| 125 | 1241-1260 | VELTSQERQCSLIEAGTRFN |  | |  |  |
| 126 | 1251-1270 | SLIEAGTRFNLGPVTITLSE |  | |  |  |
| 127 | 1261-1280 | LGPVTITLSEPRNIQQKLPP |  |  | |  |
| 128 | 1271-1290 | PRNIQQKLPPEIITLHPKIE |  |  | |  |
| 129 | 1281-1300 | EIITLHPKIEEGFFDLMHVQ |  |  | |  |
| 130 | 1291-1310 | EGFFDLMHVQKVLSASTVCK |  |  | |  |
| 131 | 1301-1320 | KVLSASTVCKLQSCTHGIPG |  |  | |  |
| 132 | 1311-1330 | LQSCTHGIPGDLQVYHIGNL |  |  | |  |
