# Supplementary material for: Epitope-mapping of the glycoprotein from Crimean-Congo hemorrhagic fever virus using a microarray approach
Source: PLoS Negl Trop Dis. 2018 Jul 9;12(7):e0006598. doi: 10.1371/journal.pntd.0006598 (PMC6053253; doi:10.1371/journal.pntd.0006598)
Supplement: S2 Table — (DOCX) [file pntd.0006598.s002.docx]

Supporting information

S2 Table, Sequences of 32 selected scan peptides in the first round that showed significant reactivity with pooled Turkish sera of CCHFV survivors.

| **ID** | **Peptide** | | **Sequence** | **ID** | **Peptide** | **Sequence** |
| --- | --- | --- | --- | --- | --- | --- |
| 1 | | p3 | ^21^TLGVLVASAAPSSPGTPGVA^40^ | 17 | p55 | ^541^ETAEIHDDNYGGPGDKITIC^560^ |
| 2 | | p10 | ^91^AGHATLREHLRDIKAENTDA^110^ | 18 | p56 | ^551^GGPGDKITICNGSTIVDQRL^570^ |
| 3 | | p14 | ^131^TSPSSSPSTPSTPQGIYHPA^150^ | 19 | p58 | ^571^GSELGCYTINRVKSFKLCEN |
| 4 | | p15 | ^141^STPQGIYHPARSLLSVSSPK^160^ | 20 | p71 | ^701^FSFGYVITCISCKALFYSLI |
| 5 | | p16 | ^151^ENIAPYKFKATMYYKDVTVS^170^ | 21 | p78 | ^771^RLTSDGLARHVTQCPKRKEK |
| 6 | | p19 | ^181^HSAMSRIPTPHTATRVSTEN^200^ | 22 | p84 | ^831^LVLLTVSLSPVQSAPVGHGK |
| 7 | | p22 | ^211^SSAQQTTPSPMTSPAQSILL^230^ | 23 | p94 | ^931^ADREIHQLHLSICKKRKTGS |
| 8 | | p23 | ^221^MTSPAQSILLMSAAPTAVQD^240^ | 24 | p95 | ^941^SICKKRKTGSNVMLAVCKRM |
| 9 | | p24 | ^231^MSAAPTAVQDIHPSPTNRSK^250^ | 25 | p99 | ^981^TTFVICILTLTICVVSTSAV^1000^ |
| 10 | | p26 | ^251^LKYNPSRVEAFHRYGTTVNC^270^ | 26 | p105 | ^1041^RKPLFLDSIVKGMKNLLNST^1060^ |
| 11 | | p27 | ^261^FHRYGTTVNCIVEEVDARSV^280^ | 27 | p107 | ^1061^SLETSLSIEAPWGAINVQST^1080^ |
| 12 | | p34 | ^331^TKARATAPTTRNLLTTPKFT^350^ | 28 | p129 | ^1281^EIITLHPKIEEGFFDLMHVQ |
| 13 | | p36 | ^351^VAWDWVPKRPSVCTMTKWQE^370^ | 29 | p130 | ^1291^EGFFDLMHVQKVLSASTVCK |
| 14 | | p37 | ^361^SVCTMTKWQEVDEMLRSEYG^380^ | 30 | p137 | ^1361^DYYCNMGDWPSCTYTGVTQH |
| 15 | | p43 | ^421^DRIFARRYNATHIKVGQPQY^440^ | 31 | p146 | ^1451^FASLACTGCYACSSGISCKV |
| 16 | | p51 | ^501^SVLRQYKTEIKIGKASTGFR^520^ | 32 | p156 | ^1551^PQSILIEHKGTIIGKQNDTC^1570^ |
