# Supplementary material for: Epitope-mapping of the glycoprotein from Crimean-Congo hemorrhagic fever virus using a microarray approach
Source: PLoS Negl Trop Dis. 2018 Jul 9;12(7):e0006598. doi: 10.1371/journal.pntd.0006598 (PMC6053253; doi:10.1371/journal.pntd.0006598)
Supplement: S3 Table — (DOCX) [file pntd.0006598.s003.docx]

S3 Table, related to Figure 2 – sequences of peptides synthesized for further epitope mapping of scan peptides #24, #55, #56, #78 and #96.

| peptide | EPITOPE WALK  scan peptide sequence | peptide | EPITOPE WALK  scan peptide sequence |
| --- | --- | --- | --- |
| EW1 | TSPAQSILLMSAAPTAVQDI | EW40 (#56) | GGPGDKITICNGSTIVDQRL |
| EW2 | SPAQSILLMSAAPTAVQDIH | EW41 | GPGDKITICNGSTIVDQRLG |
| EW3 | PAQSILLMSAAPTAVQDIHP | EW42 | PGDKITICNGSTIVDQRLGS |
| EW4 | AQSILLMSAAPTAVQDIHPS | EW43 | GDKITICNGSTIVDQRLGSE |
| EW5 | QSILLMSAAPTAVQDIHPSP | EW44 | DKITICNGSTIVDQRLGSEL |
| EW6 | SILLMSAAPTAVQDIHPSPT | EW45 | KITICNGSTIVDQRLGSELG |
| EW7 | ILLMSAAPTAVQDIHPSPTN | EW46 | ITICNGSTIVDQRLGSELGC |
| EW8 | LLMSAAPTAVQDIHPSPTNR | EW47 | TICNGSTIVDQRLGSELGCY |
| EW9 | LMSAAPTAVQDIHPSPTNRS | EW48 | ICNGSTIVDQRLGSELGCYT |
| EW10 (#24) | MSAAPTAVQDIHPSPTNRSK | EW49 | CNGSTIVDQRLGSELGCYTI |
| EW11 | SAAPTAVQDIHPSPTNRSKR | EW50 | NGSTIVDQRLGSELGCYTIN |
| EW12 | AAPTAVQDIHPSPTNRSKRN | EW51 | YNICPYCASRLTSDGLARHV |
| EW13 | APTAVQDIHPSPTNRSKRNL | EW52 | NICPYCASRLTSDGLARHVT |
| EW14 | PTAVQDIHPSPTNRSKRNLE | EW53 | ICPYCASRLTSDGLARHVTQ |
| EW15 | TAVQDIHPSPTNRSKRNLET | EW54 | CPYCASRLTSDGLARHVTQC |
| EW16 | AVQDIHPSPTNRSKRNLETE | EW55 | PYCASRLTSDGLARHVTQCP |
| EW17 | VQDIHPSPTNRSKRNLETEI | EW56 | YCASRLTSDGLARHVTQCPK |
| EW18 | QDIHPSPTNRSKRNLETEII | EW57 | CASRLTSDGLARHVTQCPKR |
| EW19 | DIHPSPTNRSKRNLETEIIL | EW58 | ASRLTSDGLARHVTQCPKRK |
| EW20 | IHPSPTNRSKRNLETEIILT | EW59 | SRLTSDGLARHVTQCPKRKE |
| EW21 | ISRTQLLRTETAEIHDDNYG | EW60 (#78) | RLTSDGLARHVTQCPKRKEK |
| EW22 | SRTQLLRTETAEIHDDNYGG | EW61 | LTSDGLARHVTQCPKRKEKV |
| EW23 | RTQLLRTETAEIHDDNYGGP | EW62 | TSDGLARHVTQCPKRKEKVE |
| EW24 | TQLLRTETAEIHDDNYGGPG | EW63 | SDGLARHVTQCPKRKEKVEE |
| EW25 | QLLRTETAEIHDDNYGGPGD | EW64 | DGLARHVTQCPKRKEKVEET |
| EW26 | LLRTETAEIHDDNYGGPGDK | EW65 | GLARHVTQCPKRKEKVEETE |
| EW27 | LRTETAEIHDDNYGGPGDKI | EW66 | LARHVTQCPKRKEKVEETEL |
| EW28 | RTETAEIHDDNYGGPGDKIT | EW67 | ARHVTQCPKRKEKVEETELY |
| EW29 | TETAEIHDDNYGGPGDKITI | EW68 | RHVTQCPKRKEKVEETELYL |
| EW30 (#55) | ETAEIHDDNYGGPGDKITIC | EW69 | HVTQCPKRKEKVEETELYLN |
| EW31 | TAEIHDDNYGGPGDKITICN | EW70 | VTQCPKRKEKVEETELYLNL |
| EW32 | AEIHDDNYGGPGDKITICNG |  |  |
| EW33 | EIHDDNYGGPGDKITICNGS |  |  |
| EW34 | IHDDNYGGPGDKITICNGST |  |  |
| EW35 | HDDNYGGPGDKITICNGSTI |  |  |
| EW36 | DDNYGGPGDKITICNGSTIV |  |  |
| EW37 | DNYGGPGDKITICNGSTIVD |  |  |
| EW38 | NYGGPGDKITICNGSTIVDQ |  |  |
| EW39 | YGGPGDKITICNGSTIVDQR |  |  |
| EW71 | ICKKRKTGSNVMLAVCKRMC |  |  |
| EW72 | CKKRKTGSNVMLAVCKRMCF |  |  |
| EW73 | KKRKTGSNVMLAVCKRMCFR |  |  |
| EW74 | KRKTGSNVMLAVCKRMCFRA |  |  |
| EW75 | RKTGSNVMLAVCKRMCFRAT |  |  |
| EW76 | KTGSNVMLAVCKRMCFRATI |  |  |
| EW77 | TGSNVMLAVCKRMCFRATIE |  |  |
| EW78 | GSNVMLAVCKRMCFRATIEA |  |  |
| EW79 | SNVMLAVCKRMCFRATIEAS |  |  |
| EW80 (#96) | NVMLAVCKRMCFRATIEASR |  |  |
| EW81 | VMLAVCKRMCFRATIEASRR |  |  |
| EW82 | MLAVCKRMCFRATIEASRRA |  |  |
| EW83 | LAVCKRMCFRATIEASRRAL |  |  |
| EW84 | AVCKRMCFRATIEASRRALL |  |  |
| EW85 | VCKRMCFRATIEASRRALLI |  |  |
| EW86 | CKRMCFRATIEASRRALLIR |  |  |
| EW87 | KRMCFRATIEASRRALLIRS |  |  |
| EW88 | RMCFRATIEASRRALLIRSI |  |  |
| EW89 | MCFRATIEASRRALLIRSII |  |  |
| EW90 | CFRATIEASRRALLIRSIIN |  |  |
|  |  |  |  |
|  |  |  |  |
