# Supplementary material for: Epitope-mapping of the glycoprotein from Crimean-Congo hemorrhagic fever virus using a microarray approach
Source: PLoS Negl Trop Dis. 2018 Jul 9;12(7):e0006598. doi: 10.1371/journal.pntd.0006598 (PMC6053253; doi:10.1371/journal.pntd.0006598)

Supporting information

S1 Fig. On-slide enzymatic O-glycosylation of CCHFV scan peptides (20mer with 10mer overlap) with recombinant GalNAcT2 and GalNAcT3. Red colored amino acids sites on peptide sequences are predicted O-glycosylation sites by NetOGlyc 3.1 algorithm. Bar graphs are representing relative fluorescence values after VVA lectin staining.

Seq.No. Scan peptide ppGalNAc-transferase 2 ppGalNAc-transferase 3


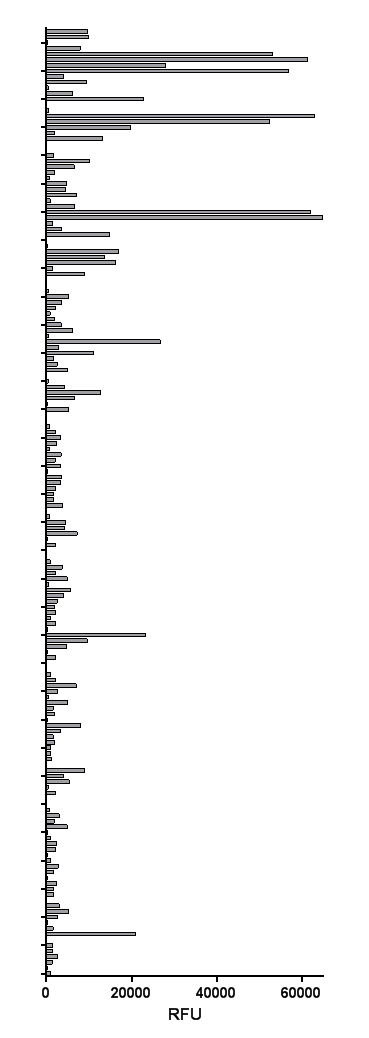

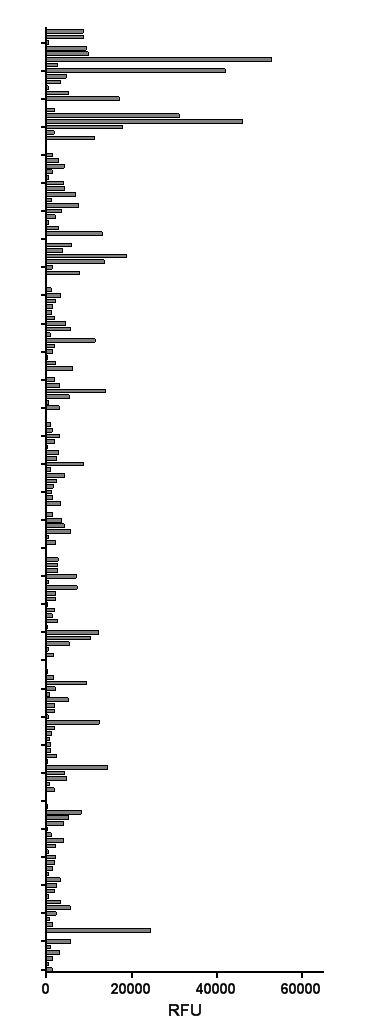

Supplement: S1 Fig — (DOCX) [file pntd.0006598.s004.docx]
